# Risk Factors Associated with Defaulted Follow-Up and Sharp Injury Management among Health Care Workers in a Teaching Hospital in Northeastern Malaysia

**DOI:** 10.3390/ijerph19116641

**Published:** 2022-05-29

**Authors:** Ahmed Farrasyah Mohd Kutubudin, Wan Mohd Zahiruddin Wan Mohammad, Siti Suraiya Md Noor, Mohd Nazri Shafei

**Affiliations:** 1 Department of Community Medicine, School of Medical Sciences, Health Campus, Universiti Sains Malaysia, 16150 Kota Bharu, Kelantan, Malaysia; ahmedfarrasyah@gmail.com (A.F.M.K.); drzahir@usm.my (W.M.Z.W.M.); 2 Department of Medical Microbiology & Parasitology, School of Medical Sciences, Universiti Sains Malaysia, 16150 Kota Bharu, Kelantan, Malaysia; ssuraiya@usm.my

**Keywords:** defaulted follow up, sharp injury, needle stick, health care worker

## Abstract

Sharp injury is a serious occupational risk for healthcare workers (HCWs). This study aimed to determine the distribution and associated factors of sharp injury cases among HCWs working at a teaching hospital in northeastern Malaysia. This was a retrospective cohort study on all reported sharp injury cases from 2015 to 2020. The secondary data were examined using descriptive and multiple logistic regression. Statistical significance was determined for associated factors of HCWs who did not attend immediate treatment after a sharp injury or any of the subsequent follow-up variables, with a *p*-value of less than 0.05. A total of 286 cases fulfilled the study criteria. The mean (SD) age of sharp injury was 29.4 (5.38) years. The overall defaulted rate for follow-up was 51.4%. Multiple logistic regression revealed a significant relationship between defaulted follow up on sharp injury management and job category as well as the type of device used. Being a doctor (Adj OR 2.37; 95% CI: 1.40, 4.03; *p* = 0.010) and those using other sharp instruments such as Coupland and drip sets (Adj OR 4.55; 95% CI: 1.59, 13.02; *p* = 0.005) had a higher odds to default follow up on sharp injury management. In conclusion, although there is a link between defaulting the follow-up and both the work category and the type of device that caused the injury, a deeper analysis is needed to uncover any additional factors and determine the appropriate intervention strategies to ensure follow up adherence.

## 1. Introduction

Sharp injury is a significant occupational health risk affecting health care workers (HCWs) [1]. Sharp injury is defined as an injury caused by a hollow bore needle or suture needle, or an injury caused by broken glass, any sharp devices, or other types of needles [2]. HCWs are always at risk of receiving sharp injuries in the workplace, and sharp injuries are a significant occupational health issue globally. The transfer of blood-borne viruses, such as HIV, hepatitis B virus (HBV), and hepatitis C virus (HCV) is the most concerning result of a sharp injury.

Globally, the prevalence of sharp injury ranged from 24.9% to 42.5%, based on the health care settings and risk [3,4,5,6]. Lee and Noor Hassim (2005) found that the prevalence of sharp injury in Malaysia was 24.9%, and the occupation with the highest risk was doctors at 48% [6]. Another study found that the risk of sharp injuries is highest among medical students [7]. Based on recent studies in Malaysia, the prevalence has dropped to a range between 0.6% and 0.8% [1,8]. The top three occupations with the highest prevalence are house officers (HO) (31.8%), nurses (17.6%), and medical officers (11.5%). The majority of the injuries occur in the ward (37.7%) and 14.9% in the operating theater [9]. 

Sharp injury is a common workplace health risk with the potentially fatal complications of bloodborne infections. Furthermore, it is a preventable condition, and ultimately everybody should be aiming to reduce and eliminate such incidences. A high number of cases will create a burden on many parties, such as HCWs, patients, and the government. HCWs who sustained the injury might develop stress, and this will affect the quality of their work, which might increase the chance of another sharp injury occurrence. If the HCWs contracted a blood-borne infection as a result of a sharp injury, they will need to begin treatment, and this will add to the country’s financial burden [10,11]. HCWs who become infected are more likely to take medical leave, hence reducing human resources. Human capital, healthcare organizations, and the entire system can all be impacted by a single traumatic injury. Many studies have revealed that HCWs did not complete the post-exposure follow-up despite being contacted by phone and mail [12]. The percentage of HCWs who completed post-exposure follow up dropped as time passed after the accident. However, the pattern of sharp injury cases and follow up practices indicated an improved tendency to attend a follow-up [13]. The rate of defaulted follow-up for sharp injuries in Malaysia was 28%, and the global prevalence of defaulted follow-ups ranges from 28% to 53% [1,14].

There have been several studies looking at the factors associated with sharp injury, but there have been few studies that look at the factors associated with defaulted follow-up for sharp injury management among HCWs, both internationally and regionally. This is very alarming, as apart from notifying authorities in the event of a sharp injury, HCWs must also follow up to ensure that any infection that resulted from the injury does not spread. If follow-up was maintained, seroconversion status and other consequences could be detected sooner, allowing for immediate treatment. This study will determine the prevalence and the risk factors associated with defaulted sharp injury follow-ups, with the aim of providing insight that can be employed in future interventional studies. The outcomes of this study could aid stakeholders in establishing effective prevention initiatives in clinical practice. 

## 2. Materials and Methods

### 2.1. Study Design and Population

This was a retrospective cohort study of 286 sharp injury cases among HCWs working at Hospital Universiti Sains Malaysia (USM) obtained from the Sharp Injury Incidence Form database from 2015 to 2020. The form was designed by the institution’s infection control and hospital epidemiology unit. HCWs who reported the injury for the first time between 1st January 2015 and 31st December 2020 were included in the study. The required sample size was calculated using a single proportion formula based on the prevalence of defaulted follow up for sharp injuries among HCWs (36%) [13]. Considering that 10% of the data were incomplete, the required sample size was 389. However, there were only 286 participants that satisfied the study criteria. As a result, no sampling strategy was applied because the current study included all HCWs who satisfied the study criteria. There were different sections in this form, which consisted of sociodemographic, infection source, event history, and follow-ups. HCWs receive treatment and follow-up after suffering a sharp injury. Depending on the nature of the situation, the follow-up may be given up to five times. After the incident, participants can be followed up at one week, six weeks, three months, six months, and one year after the injury (for high-risk situations). 

### 2.2. Data Collection and Research Tool

The Sharp Injury Incidence Form was personally checked to ensure correct entry and data completeness, reducing the risk of error. In the database used for this study, sharp injury refers to any injury caused by any sharp instrument used in a health care facility, which includes all sorts of needles, scalpels, trocar, shattered glass, and other sharp devices [2].

In this study, HCW refers to any person who works in health care settings and institutions, and frequently comes into touch with patients, blood, and other bodily fluids. Medical consultants, specialists, medical officers, HOs, nurses, dentists, and pharmacists are all considered HCWs in this study [2]. Outsourced employees, students, patients, relatives, and visitors were excluded due to the low levels of exposure to needles and sharp-related procedures in their specific roles; their exclusion could result in heterogeneity in the study population’s features. In the present study, if HCWs did not attend a prompt treatment after a sharp injury or any of the subsequent follow-ups, they were deemed to have defaulted follow-up for sharp injury management [2]. More than 20% of the responses were discarded because the data were incomplete. The Sharp Injury Incidence Form responses were categorized and coded. Data was double-checked by returning to the form if there were any questions. To transfer data from the Sharp Injury Incidence Form to IBM SPSS statistic version 26 for statistical analyses, a proforma was used.

### 2.3. Data Analyses

IBM SPSS statistic version 26 was used for data entry and analysis. Once the data was entered, it was examined, checked, and cleaned. A preliminary data description was performed to find any missing data and values. In the descriptive analysis, data were analyzed and presented as frequencies and percentages. Bivariable data were explored using a simple logistic regression to evaluate if there was a link between the defaulted follow-ups and other individual- and injury-specific variables, such as sociodemographic and job-related variables, and the cause and type of the injury. A crude odds ratio (OR) and a *p*-value were used to represent the findings. 

Multiple logistic regression analyses were used to find the predictors of the defaulted follow-ups. All independent variables were initially included. After analyzing the model using Forward LR and Backward LR approaches, a tentative main effect model was developed. There were no signs of interaction or multicollinearity. 

Additionally, all two-way interactions were investigated. Following this, the preliminary final model was built. The model’s fitness was assessed using Hosmer and Lemeshow’s goodness-of-fit test. The classification table and receiver operating characteristic (ROC) curve were also used to determine the model’s fitness. The results were presented as a *p*-value and an adjusted OR. The significance level was set at a *p*-value of less than 0.05.

### 2.4. Ethical Issues Consideration

Data collection began after receiving approval from the Universiti Sains Malaysia Human Research Ethics Committee on 13 December 2020 (USM/JEPeM/20110583). Response anonymity was used to ensure data confidentiality, and only the researchers had access to the data.

## 3. Results

From 2015 to 2020, there were 286 sharp injury cases involving contaminated or presumed contaminated objects that required follow up at one week, six-week, three-month, six months, and one year (for high-risk cases) after the incident. The mean (SD) age of the participants was 29.4 (5.38) years. Most of them were female (65.0%), and the majority were Malays (85.7%). The highest number of cases occurred in the ward/in-patient (55.9%). The majority of them occurred in the surgical based facilities (22.4%), were among House officers (38.8%), occurred due to branula and needle (72.4%), and 98.6% of the devices were contaminated. Most of them happened while handling patients (40.0%), and the majority of the contamination source was known (95.1%). Among all participants who were given follow up, the majority of them (51.4%) defaulted follow-up at one point in time. The above-mentioned data is presented in Table 1. 

**Figure 1:**
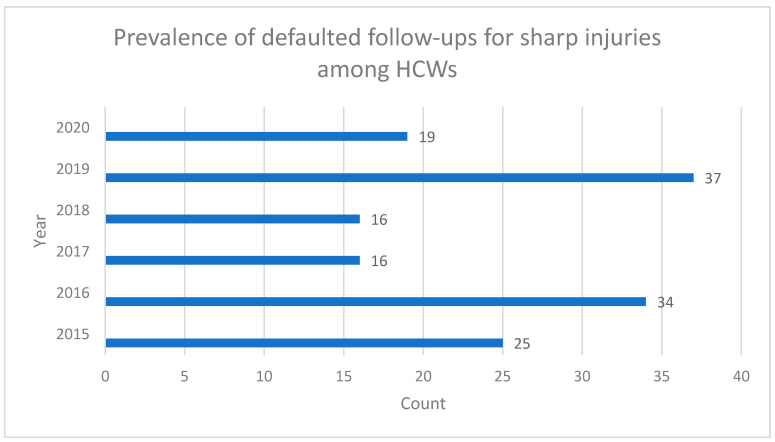
Prevalence of defaulted follow ups for sharp injuries among HCWs by year (n = 147).

Figure 1 shows all the defaulted cases and their year of occurrence. The highest number of follow up defaulters was found to be in the year 2019 (37 cases).

Table 2 shows the studied variables that might have been associated with defaulting a follow-up after a sharp injury among HCWs. It was found that the job category and the type of device were statistically significant in their association with the defaulted follow ups.

## 4. Discussion

In this study, several HCWs did not complete the post-exposure follow-up management. We also discovered that HCWs’ compliance with post-exposure follow-up was associated with a number of the characteristics stated previously. The employer was responsible for ensuring a safe working environment, warning all employees about the danger of infection, and mandating that all injuries are recorded and documented, including mucocutaneous exposure to patients’ bodily fluids. Inadequate follow-up can lead to an illness that goes unnoticed, posing a serious risk to the employee, as well as future patients. This should not happen and the patient should be protected from this risk as part of the clinical risk management. Clinical risk management is a structured response plan to identify conditions that put patients at risk of injury, and then take steps to prevent or reduce those risks by enhancing the quality and safety of healthcare services [15]. A phone call was reported to be the most prevalent strategy (85.7%) for ensuring follow-up enforcement [16].

In this study, we found that more than half (51.4%) of all sharp injury cases had defaulted follow-up either at one week, three weeks, three months, or six months after having been injured. Globally, the prevalence of defaulted follow-ups for sharp injuries ranges from 28% to 53% [1,14]. In Malaysia, based on a study conducted in Selangor, the prevalence of defaulted follow-ups for sharp injuries is 27.7%, which is significantly lower than the findings of this present study [1]. This demonstrated that the findings from this study were clearly higher than average. This study was done in a teaching hospital, therefore the setting might be different than the previous studies that have been undertaken in Malaysia. This study’s population were only from a single teaching hospital, which might be limited in terms of diversity. Previous studies were completed in multiple hospitals. 

The present study illustrated that there was an association between job category and defaulted follow-ups among HCWs. According to this study, the HCWs who had the highest numbers of sharp injuries were among HOs [111 (38.8%)]. A total of 184 (64.3%) doctors had a sharp injury. Nearly 75% of doctors failed to follow up on a sharp injury, with HOs of the most likely to failing to do so (45.6%). This was a staggering amount when compared to other occupations such as nurses, medical assistants, pharmacists, dentists, health care assistants, and laboratory technicians. This finding was supported by a few other studies. A previous study found that there was a statistically significant association between defaulted follow up and occupational status and category [17,18].

Doctors should have a better understanding of the necessity of treatment and follow up procedures after experiencing a sharp injury, as they are likely to be educated in bloodborne diseases, and infection risk and transmission. As a result, doctors should be the most compliant of all job categories. Being a doctor, on the other hand, was linked to defaulting follow-ups in this study. It isn’t always advantageous to have the most up-to-date information. The adage “the more the better” may not necessarily apply when it comes to information. There was an “inverted U” link between comprehension and enforcement in some cases [19,20].

One possible explanation for this so-called highly educated patient’s non-compliance with their care is that when people know too much, they prefer to self-manage and self-treat, making them feel less compelled to attend follow up appointments or proceed with treatment. When they have more knowledge and experience, they tend to self-treat and self-manage. This could be because they believe they have sufficient information to self-diagnose, self-treat, and self-medicate without adhering to treatment and follow-up. Those with lesser knowledge were less likely to self-treat and self-manage, and were more likely to be compliant with treatment and follow up procedures [19,20,21,22].

Apart from possessing the knowledge, another reason why doctors choose to self-treat and self-manage is to save time. They use the knowledge to self-treat to save time, rather than attending their appointments and following up [23,24]. Normally, doctors didn’t simply default without treatment. They can easily ask another doctor to check on them, take a serologic investigation, and trace the result by themselves. This process was quicker and time-saving, and had the same result and desired outcome without having to spend time following up.

In this study, the greatest number of injuries occurred when dealing with branula and needles (72.4%). The highest number of defaulters had their injury caused by branula and needles (72.1%). We discovered, therefore, that there was a statistically significant association between the type of device that caused the injury and defaulting on the follow-up procedures. HCWs who sustained a sharp injury because of surgical instruments and other sharp objects were more likely to default compared to injury caused by branula and needles. 

HCWs who were injured by surgical instruments were more likely to default because they knew the patients’ serologic status before they were injured. Almost all patients undergo a serological investigation prior to going into surgery. The existence of virus-specific IgM antibodies, or a large rise in the levels of specific IgG antibodies, is used to make a serological diagnosis. When HCWs sustained a sharp injury, they already knew the serology and virology status of the patient. Normally, most of the patients who undergo surgical procedures are free from bloodborne pathogens. Therefore, in incidences where contaminated surgical instruments have caused the injury, and the source was known to be pathogen-free, HCWs knew for sure that they did not have any risk of receiving a blood-borne infection, such as HIV, HCV, or HBV. They were assured and had no reason to worry. This will lead them to think that they have a reduced need to seek treatment and attend follow ups after a sharp injury. 

Another reason that HCWs tend to default after they have been injured by surgical instruments is the surgical glove’s protection. Bloodborne pathogens can be transmitted to HCWs by breaches in the glove barrier during surgical procedures. Surgical gloves are known to act as a mechanical barrier against communicable diseases [25,26]. This study found that HCWs who sustained sharp injuries due to other sharp objects were at even higher odds to default. In this study, the other sharp devices were the knife, Coupland, drip sets, periodontal and endodontic probe tip, guidewire, monopolar pin, probes, Steinmann pin, bone fragment, and suprapubic catheter. The majority of the devices in this category are instruments that are used by dentists. Such instruments that are used by dentists tend to be sterile. During dental procedures, the tools used will be contaminated with blood, body fluids, and other contaminants, which will be cleaned and sterilized using various sterilization methods [27]. Even though dental surgery is not as complicated as medical surgery, it still necessitates a high level of infection control and safety practices to prevent cross-contamination and occupational exposure to blood-borne infections. Hence, the risk of infection between patients and dentists is reduced [28]. 

This study has a few limitations. This study was a retrospective cohort study that relied on secondary data. It was hampered by a lack of information. Furthermore, if the variable or record used was not intended for research, data availability may be limited. There was also a possible absence of data on potential confounding factors because the data was recorded in the past. It also has the risk of information bias due to recall bias. Examples of recall bias were contamination source, type of device, and the procedure conducted. 

Some instances had to be omitted from the study due to the inclusion and exclusion criteria, lowering the sample size and perhaps affecting the power of the study. The desired sample size was significantly larger than the available sample size, and exclusion rules reduced the sample size even further. As a result, one of the studied factors had a low power with large confidence intervals. 

In the future, we recommend that a study focusing on the variables associated with defaulted follow-ups for sharp injuries should be expanded to the entire country to define the exact problems, burdens, and common important predictors that cause such injuries. Understand the causes can help us in addressing the sharp injury problem and reducing it to zero. The future study should also include other important variables, which are not included in the present study. Factors such as duration of employment, duration of reporting, and duration of hours at work are not available in the database, but proved to be important predictors. The authors of this study believe that studying these variables may help achieve sharp injury prevention goals. We also recommend that future research takes into account mucocutaneous and skin exposures to blood and bodily fluid, as this also poses a risk of bloodborne infection transmission to HCWs. In addition, future research is recommended to include a qualitative approach to understand the reasons for defaulting sharp injury follow ups, and also determine if it varies by job category or setting. It is also recommended for all HCWs to update their knowledge of sharp injuries, prevention, and treatment, so that such injuries and defaulted follow-ups can be reduced.

## 5. Conclusions

The prevalence of defaulted follow-up for sharp injuries among HCWs was relatively high in northeastern Malaysia. There is a significant association between defaulted follow-ups and both work category and the type of device that caused the injury. Hence, a deeper analysis is needed to uncover any additional factors and determine the appropriate intervention strategy to ensure follow up adherence. 

We recommend conducting multi-center studies in Malaysia with a bigger sample size using mixed techniques to improve precision, and to reveal plausible reasons for defaulted follow-up for sharp injuries, allowing for improved planning and execution of public health preventive interventions at all levels. Despite its flaws, this study represents an important step toward preventing sharp injuries and ensuring follow-up compliance.

## Figures and Tables

**Table 1:** Characteristics of sharp injury cases among HCWs in Hospital USM and follow up distribution (n = 286).

Variables	n (%)	Defaulted (n = 147)	Completed (n = 139)
		n (%)	n (%)
Age (years)			
<25	23 (8.1)	8 (5.4)	15 (10.8)
25–29	152 (53.1)	82 (55.8)	70 (50.4)
30–34	68 (23.8)	39 (26.5)	29 (20.8)
≥35	43 (15.0)	18 (12.3)	25 (18.0)
Gender			
Female	186 (65.0)	94 (63.9)	92 (66.2)
Male	100 (35.0)	53 (36.1)	47 (33.8)
Ethnicity			
Malay	245 (85.6)	125 (85.0)	120 (86.3)
Chinese	30 (10.5)	15 (10.2)	15 (10.8)
Indian	11 (3.9)	7 (4.8)	4 (2.9)
Job Category			
House officer	111 (38.8)	67 (45.6)	44 (31.7)
Medical officer	62 (21.7)	37 (25.2)	25 (18.0)
Specialist	11 (3.8)	6 (4.0)	5 (3.6)
Nurse	66 (23.2)	26 (17.7)	40 (28.8)
Dentist	17 (5.9)	5 (3.4)	12 (8.6)
Others	19 (6.6)	6 (3.1)	13 (9.3)
Location of Event			
Outpatient clinic	25 (8.7)	12 (8.2)	13 (9.4)
Ward/inpatient	160 (55.9)	80 (54.4)	80 (57.6)
A&E	29 (10.2)	17 (11.6)	12 (8.6)
OT	56 (19.6)	32 (21.8)	24 (17.2)
Others	16 (5.6)	6 (4.0)	10 (7.2)
Department			
Multidisciplinary	64 (22.4)	35 (23.8)	29 (20.9)
Medical based	104 (36.4)	53 (36.1)	51 (36.7)
Surgical Based	118 (41.2)	59 (40.1)	59 (42.4)
Type of Device			
Branula & needle	207 (72.4)	106 (72.1)	101 (72.7)
Surgical Instrument	55 (19.2)	34 (23.1)	21 (15.1)
Others	24 (8.4)	7 (4.8)	17 (12.2)
Device Contamination			
Yes	282 (98.6)	147 (100.0)	135 (97.1)
No	4 (1.4)	0 (0.0)	4 (2.9)
Procedure Conducted			
Handling patient	116 (40.6)	57 (38.8)	59 (42.4)
Handling equipment	64 (22.4)	30 (20.4)	34 (24.5)
Disposal related	46 (16.1)	22 (15.0)	24 (17.3)
Inoperative field	43 (15.0)	28 (19.0)	15 (10.8)
Others	17 (5.9)	10 (6.8)	7 (5.0)
Contamination Source			
Known	272 (95.1)	141 (95.9)	131 (94.2)
Unknown	14 (4.9)	6 (4.1)	8 (5.8)

**Table 2:** Variables associated with defaulting the sharp injury follow-up among HCWs using simple and multiple logistic regression analyses (n = 286).

Variables	Defaulted (n = 147)	Crude OR ^a^ (95% CI)	Adjusted OR ^b^ (95% CI)	*p*-Value
Age (years)				
≥40	5/12	1		
<40	142/274	1.06 (0.39, 2.91)		
Gender				
Female	94/186	1		
Male	53/100	1.10 (0.68, 1.80)		
Ethnicity				
Non-Malay	22/41	1		
Malay	125/245	0.90 (0.46, 1.75)		
Job Category				
Non-medical doctor	37/102	1	1	
Medical Doctor	110/184	2.22 (1.32, 3.73)	2.38 (1.40, 4.03)	0.001
Location of Event				
Outpatient	12/25	1		
Ward/inpatient	129/245	1.93 (0.62, 6.07)		
Others	6/16	1.80 (0.63, 5.11)		
Department				
Multidisciplinary	35/64	1		
Medical based	53/104	0.86 (0.46, 1.61)		
Surgical based	59/118	0.83 (0.45, 1.53)		
Type of Device				
Branula & needle	106/207	1	1	
Surgical Instrument	34/55	2.55 (1.01, 6.41)	2.96 (1.16, 7.53)	0.023
Others	7/24	3.93 (1.40, 11.07)	4.55(1.59, 13.02)	0.005
Procedure Conducted				
Disposal related	32/63	1		
Handling patient	57/116	1.04 (0.50, 2.14)		
Handling equipment	58/107	1.27 (0.66, 2.45)		
Contamination Source				
Unknown	6/14	1		
Known	141/272	1.44 (0.49, 4.25)		

^a^ Simple Logistic Regression. ^b^ Multiple Logistic Regression. Constant = −1.63. Forward LR and Backward LR Multiple Logistic Regression was applied. No multicollinearity and no interaction. Hosmer–Lemeshow test, *p*-value = 0.991. Classification Table (overall correctly classified percentage = 65.1%). Area under the curve = 63.3%.

## Data Availability

There is no reported data.
